# Adipose-derived stem cell spheroid-laden microbial transglutaminase cross-linked gelatin hydrogel for treating diabetic periodontal wounds and craniofacial defects

**DOI:** 10.1186/s13287-023-03238-2

**Published:** 2023-02-03

**Authors:** Che-Chang Tu, Nai-Chen Cheng, Jiashing Yu, Yi-Xuan Pan, Wei-Chiu Tai, Yin-Chuan Chen, Po-Chun Chang

**Affiliations:** 1 Graduate Institute of Clinical Dentistry, School of Dentistry, College of Medicine, National Taiwan University, Taipei, Taiwan; 2 Division of Periodontics, Department of Dentistry, National Taiwan University Hospital, Taipei, Taiwan; 3 Department of Surgery, National Taiwan University Hospital and College of Medicine, Taipei, Taiwan; 4 Department of Chemical Engineering, College of Engineering, National Taiwan University, Taipei, Taiwan; 5 School of Dentistry, College of Dental Medicine, Kaohsiung Medical University, Kaohsiung, Taiwan

**Keywords:** Mesenchymal stem cell, Diabetes, Wound healing, Tissue engineering, Bone regeneration, Hydrogels

## Abstract

**Background:**

Diabetes mellitus deteriorates the destruction and impairs the healing of periodontal wounds and craniofacial defects. This study is to evaluate the potential of self-assembled adipose-derived stem cell spheroids (ADsp) in microbial transglutaminase cross-linked gelatin hydrogel (mTG) for treating diabetic periodontal wounds and craniofacial defects.

**Methods:**

Human adipose-derived stem cells (ADSCs) were isolated by lipoaspiration, pluripotent genes and trilineage differentiation were examined, and the maintenance of ADsp properties in mTG was verified. Oral mucosal wounds and calvarial osseous defects were created in diabetic rats. Gross observation, histologic evaluation, and immunohistochemistry for proliferating cells and keratinization were conducted in the mucosal wounds within 4–28 days. Micro-CT imaging, histologic evaluation, and immunohistochemistry for proliferating cells and osteogenic differentiation were conducted in the osseous defects at 7 and 28 days.

**Results:**

ADSCs expressed pluripotent genes and were capable of trilineage differentiation. ADsp retained morphology and stemness in mTG. In diabetic mucosal wounds, wound closure, epithelization, and keratinization were accelerated in those with ADsp and ADsp-mTG. In diabetic osseous defects, osteogenic differentiation markers were evidently expressed, cell proliferation was promoted from day 7, and bone formation was significantly promoted at day 28 in those with osteogenically pretreated ADsp-mTG.

**Conclusions:**

ADsp-mTG accelerated diabetic oral mucosal wound healing, and osteogenically pretreated ADsp-mTG promoted diabetic osseous defect regeneration, proving that ADsp-mTG facilitated diabetic periodontal wound healing and craniofacial osseous defect regeneration.

## Introduction

Diabetes mellitus (DM) affected 463 million people worldwide in 2019, and approximately USD 760 billion in global health expenditure was spent on treating DM [1]. DM comprises metabolic disorders characterized by hyperglycemia related to inadequate insulin production or improper response of the cells to insulin, and the major complications include poor wound healing, cardiovascular diseases, retinopathy, and nephropathy [2]. As hyperglycemia causes the shift in subgingival microbiota and the accumulation of advanced glycation end-products (AGEs) activates receptor for AGE (RAGE) to increase proinflammatory cytokine secretion in the periodontium [3–5], periodontal destruction deteriorates, wound healing is retarded, and the outcome of periodontal regeneration is impaired [6, 7]. The American Diabetes Association defined periodontal disease as a common comorbidity of DM in 2018 [8].

Stem cell therapy has been proposed to regenerate bony defects in compromised environments [9]. Adipose-derived stem cells (ADSCs), isolated by liposuction, can be collected in large quantities with low donor site morbidity and a high yield of stem cells and have shown potential as a promising source of stem cells [10]. Previous evidence supports the capability of ADSCs to regenerate skin and osseous defects and treat wounds with inferior healing potential [11–13].

One critical aspect of stem cell therapy is to maintain stemness. While signals from the microenvironment play important roles in regulating the differentiation capability of stem cells [14], the establishment of self-assembled cell spheroids to mimic native cellular morphology, cell–cell contact, and cell–extracellular matrix interactions in a three-dimensional (3D) microenvironment has been proven to retain the properties of ADSCs [15]. Gelatin-based hydrogels appeared to be suitable vehicles to deliver ADSC spheroids (ADsp) in vivo because they are biocompatible and biodegradable and can rapidly absorb biological fluid to occupy defects. However, the cross-linking process, frequently achieved by UV irradiation or chemical reagents, exhibits certain cytotoxicity and might influence the viability of encapsulating cells [16, 17]. A recent study reported that microbial transglutaminase (mTG)-crosslinked gelatin hydrogels showed low cytotoxicity, preserved the stemness of ADsp, and exhibited excellent injectability and tissue response [18].

The hypothesis of this study was that ADsp embedded in mTG (ADsp-mTG) promoted the healing of diabetic periodontal wounds. Experimental gingival mucosal wounds and calvarial osseous defects of rats were created to represent the mucosal and osseous compartments of the periodontium and craniofacial defects. The outcome could serve as a reference for ADSC application in the diabetic periodontal wound and craniofacial defect regeneration.

## Methods

Information on the manufacturers of materials, formulations of media, mucosal wound healing indices, the conditions of real-time PCR, the sequence information of primers and probes, and protocols of immunohistochemical staining is listed in the Appendix.

### Ethical statements

The harvest of ADSCs from humans was conducted in accordance with the Declaration of Helsinki in 1975, as revised in 2013 [19], and approved by the Institutional Review Board of National Taiwan University Hospital (NTUH) under protocol no. 201303038RINB. The animal study was conducted in accordance with Basel Declaration in 2010 and was approved by the Animal Care and Use Committee of National Taiwan University under protocol no. 20190195.

### Characterization of ADSCs

#### Isolation of ADSCs

ADSCs were isolated from the subcutaneous fat tissue of four non-smoking, non-diabetic female patients (aged 32–57 years, BMI 21.0–26.6) planned to receive lipoaspiration for aesthetic plastic surgery at NTUH as previously described [20], and all of them signed informed consent prior to the harvest procedure. Following lipoaspiration, subcutaneous adipose tissue was obtained, extensively washed, and red blood cells were lysed. The remaining adipose tissue was digested with collagenase, followed by filtration and centrifugation to obtain the stromal vascular fraction (SVF). Cells in the SVF were subsequently incubated at 37 °C with 5% CO_2_ under 99% humidity and cultured in a growth medium. The medium was renewed every 2–3 days and passaged until 90% confluence was reached, and cells at passage 3 were used for all subsequent assessments. According to the results from our previous investigation, > 99% of cells were positive for stem cell surface markers including CD44, CD73, CD90, and CD166, and showed adipogenic and osteogenic potential [20].

#### Expression of pluripotent markers

The expression levels of pluripotency-associated transcription factors, including SRY-box transcription factor-2 (Sox2), octamer-binding transcription factor-4 (Oct4), core-binding factor alpha-1 (Cbfa1), and Nanog homeobox, in ADSCs were analyzed. A human foreskin fibroblast cell line (Hs68) was used as the negative control, and an induced bone marrow mesenchymal stem cell line (iBMSC) was used as the positive control. All examined cells were seeded on 6-well dishes at 1 × 10^5^ cells/well and incubated with a growth medium for 24 h. RNA was isolated using an RNA isolation kit and reversely transcribed to cDNA using a cDNA synthesis kit to run real-time PCR, and the TaqMan probes of GAPDH (housekeeping gene) and pluripotent markers were used. The level of gene expression was calculated using the comparative CT method according to the level of GAPDH and was further normalized to the expression level of Hs68. All experiments were performed in triplicate.

#### Trilineage differentiation

To evaluate the capability of adipogenic, osteogenic, and chondrogenic differentiation, cells (Hs68, iBMSCs, and ADSCs) were cultured in adipogenic, osteogenic, and chondrogenic induction medium. After 2 weeks, the cell monolayer was fixed in 4% paraformaldehyde and stained with Oil Red to observe lipid droplets for adipogenesis, stained with Alizarin Red to observe mineralized matrix apposition for osteogenesis, and stained with Alcian Blue to observe cartilage-specific proteoglycans for chondrogenesis.

### Preparation and characterization of ADsp-mTG

#### Preparation of ADsp-mTG

To form ADSC spheroids (ADsp), the agarose microwell plates were immersed in 75% ethanol for 1 h and transferred to PBS before use. Each agarose microwell plate was placed into a 12-well plate with growth medium, and the entrapped bubbles were removed by centrifugation. ADSCs were seeded at 1 × 10^5^ cells/well, centrifuged to ensure that ADSCs were captured in the microwells, and then incubated for 3 days at 37 °C in 5% CO_2_ with 1 mL growth medium/plate. After ADsp were formed, ADsp were detached and passed through a cell strainer to collect uniform ADsp. Gelatin was dissolved in growth medium at 70ºC, followed by sterile filtration through 0.22-µm filters. A 1 mL/well gelatin/microbial transglutaminase solution (6% gelatin, 60 U g^−1^ microbial transglutaminase) mixed with ADsp was loaded to achieve a final density of 1.2 × 10^6^ cells/100 μL (approximately 3072 ADsp/100 μL).

#### Behaviors of ADsp in mTG

The morphology and dynamic changes of ADsp in the medium and mTG were observed under a light microscope on days 1–4. The area and Feret diameters of ADsp were measured using ImageJ. The trilineage differentiation of ADsp in mTG was assessed by loading ADsp-mTG into 6-well plates, which were incubated with the appropriate induction medium for 14 days and stained as described in “Trilineage differentiation” section.

### Preclinical validation

#### The induction of diabetes and randomization

Male Sprague–Dawley rats (weighing 250 g) were purchased from BioLASCO Taiwan Co. Ltd. (Taipei, Taiwan) and were housed in a climate-controlled room (21 °C) on a 12:12-h light/dark cycle with free access to food and water. Several of them received a one-time intraperitoneal injection of streptozotocin (STZ) (65 mg/kg) diluted in citrate buffer (0.05 M, pH 4.5) to induce diabetes. Fasting blood glucose (FBG) was checked using a glucose meter, and glycated hemoglobin (HbA1c) was checked using an HbA1c analyzer before and after 3 weeks of induction [7]. FBG > 250 mg/dL was considered diabetic. Rats without STZ injection (ND rats) served as controls, and FBG and HbA1c were checked at the time of wound/defect creation.

#### The mucosal wound model

**Fig. 1:**
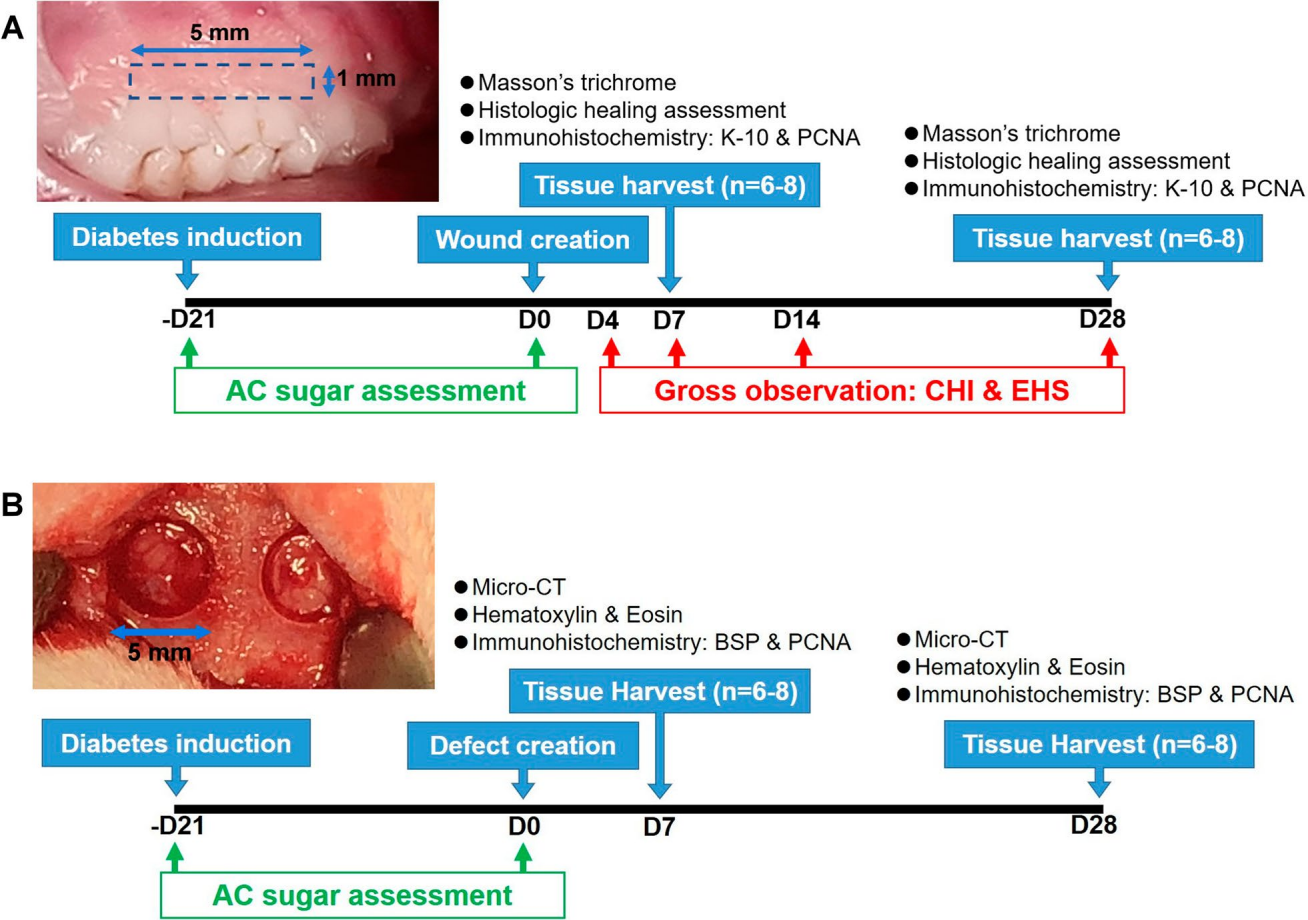
Designs of animal models. **A** The mucosal wound model. **B** The osseous defect model

The design of the model is illustrated in Fig. 1A. Twenty-four rats with successful diabetes induction (DB) and 16 ND rats were utilized. The surgical procedures were covered by general anesthesia using an intraperitoneal injection of 0.1 mL/kg zolazepam–tiletamine and 0.5 mL/kg xylazine. In DB rats, bilateral 1 × 5 mm full-thickness gingival wounds on the palatal gingival margin of the maxillary first and second molars were surgically created, and the wounds were randomly assigned to the following treatment: untreated (DB-CL), ADsp alone (DB-AS), or ADsp-mTG (DB-AT). The randomization was determined by a simple draw by a blinded administrator and was minorly adjusted to ensure that the contralateral site received a different treatment assignment, and in each wound, 10 µL hydrogel was applied to ensure complete coverage. In ND rats, gingival wounds were created unilaterally without any further treatment (ND-CL). Animals were euthanized using carbon dioxide on days 7 and 28 (n = 8/treatment/time point), and the maxillae containing the examined wounds were harvested upon euthanasia.

#### Gross observation of mucosal wounds

The photographs of mucosal wounds were taken using a digital camera on days 4, 7, 14, and 28. The healing status was assessed using the clinical healing index (CHI) and early wound healing score (EHS) [21, 22].

#### Histologic and immunohistochemical assessments of mucosal wounds

All harvested specimens were fixed in 10% buffered formalin for 3 days and then decalcified with 12.5% EDTA (pH 7.4) for 4 weeks, embedded in paraffin, and cut into 5-mm-thick sections. Masson’s trichrome staining was performed to outline the dynamics of wound healing. Histologic healing was divided into the following categories: no epithelization with a sloughing surface; full epithelization but with an epithelial cleft and reduced thickness; and full epithelization with normal consistency and contour. The signs of keratinization and cell proliferation were further evaluated by immunohistochemical staining for cytokeratin-10 (K-10) and proliferating cell nuclear antigen (PCNA). Cells expressing PCNA in the mucosal wounds were quantified in three randomly selected areas per slide at 400 × magnification, and the results were presented as the ratio of PCNA-positive cells to the total number of cells in the investigated area. All stained slices were observed under a light microscope, and images were acquired using a digital camera equipped with an imaging system.

#### The osseous defect model

The design of model is illustrated in Fig. 1B. A total of 56 animals, including 40 DB rats and 16 ND rats, were utilized in this part. The surgical procedures were covered by general anesthesia as described in “The mucosal wound model” section. Following a midline incision and blunt dissection of underlying tissue, the calvarium was exposed. Bilateral 5-mm-diameter cylinder calvarial defects were surgically created using a dental trephine bur. The defects were randomly assigned to the following treatments: untreated (DB-CL), ADsp alone (DB-AS), ADsp-mTG (DB-AT), pretreated ADsp alone (DB-PAS), or pretreated ADsp-mTG (DB-PAT). The defects were minorly adjusted to ensure that the contralateral site received a different treatment assignment. As a previous investigation reported that alkaline phosphatase activity, an early sign of osteogenesis, was significantly elevated by incubating ADSCs in the osteoinductive environment for 5–7 days [23], to direct the differentiation of ADSC toward osteogenic lineage, ADSC pretreatment was performed by incubating ADSCs in the osteogenic induction medium for 10 days before forming spheroids. The osteogenic differentiation level of the pretreated ADSCs was examined by the Alizarin Red assay described in “Trilineage differentiation” section and was quantified using an ELISA reader at a wavelength of 405 nm. In each defect, 20 µL hydrogel was applied to ensure complete coverage. In ND rats, the defects were created unilaterally without any further treatment (ND-CL). Wounds were closed by reflex wound clips. Animals were euthanized on days 7 and 28 (n = 8/treatment/time point), and the calvarias containing osseous defects were harvested upon euthanasia.

#### Micro-CT assessments of the osseous defects

The harvested calvarias were fixed in 10% buffered formalin for 3 days and then examined by a micro-CT scanner with a voxel size of 18 μm. The entire osseous defects were selected as the region of interest (ROI). The micromorphometric bone parameters within the ROI, including relative bone volume (BV/TV), trabecular thickness (Tb. Th), and trabecular number (Tb.N), were calculated using a micro-CT image analysis software.

#### Histologic and immunohistochemical assessments of the mucosal wounds

After micro-CT imaging, the specimens were processed as described in “Histologic and immunohistochemical assessments of mucosal wounds” section and were stained with hematoxylin and eosin for descriptive histology. Histomorphometric analysis was performed using ImageJ, and new bone (NB) bridging, defined as the percentage of linear bone formation to the entire defect length, and defect fill, defined as the percentage of NB area to the entire defect area, were assessed.

The signs of osteogenic differentiation and cell proliferation in the defects were further evaluated by immunohistochemical staining of bone sialoprotein (BSP) and PCNA. Cells expressing PCNA in the osseous defects were quantified in three randomly selected areas, two at the borders and one at the center of the defect, at × 400 magnification. The assessment protocol and data presentation were the same as described in “Histologic and immunohistochemical assessments of mucosal wounds” section.

### Statistical analysis

Statistical analysis was performed using statistical software. Unpaired t test was used to compare the concentration of Alizarin Red between the cultures of unpretreated and the cultures of pretreated ADSCs in vitro. Paired t-test was used to compare the levels of AC sugar and HbA1c before and after STZ injection. In DB animals, one-way ANOVA and Tukey’s post hoc tests were used to compare the differences among the treatment assignments. For CHI and EHS, a nonparametric Kruskal–Wallis test was used. The data are expressed as the means ± standard deviation, with a *p* value of less than 0.05 considered statistically significant.

## Results

### Characterization of ADSCs

**Fig. 2:**
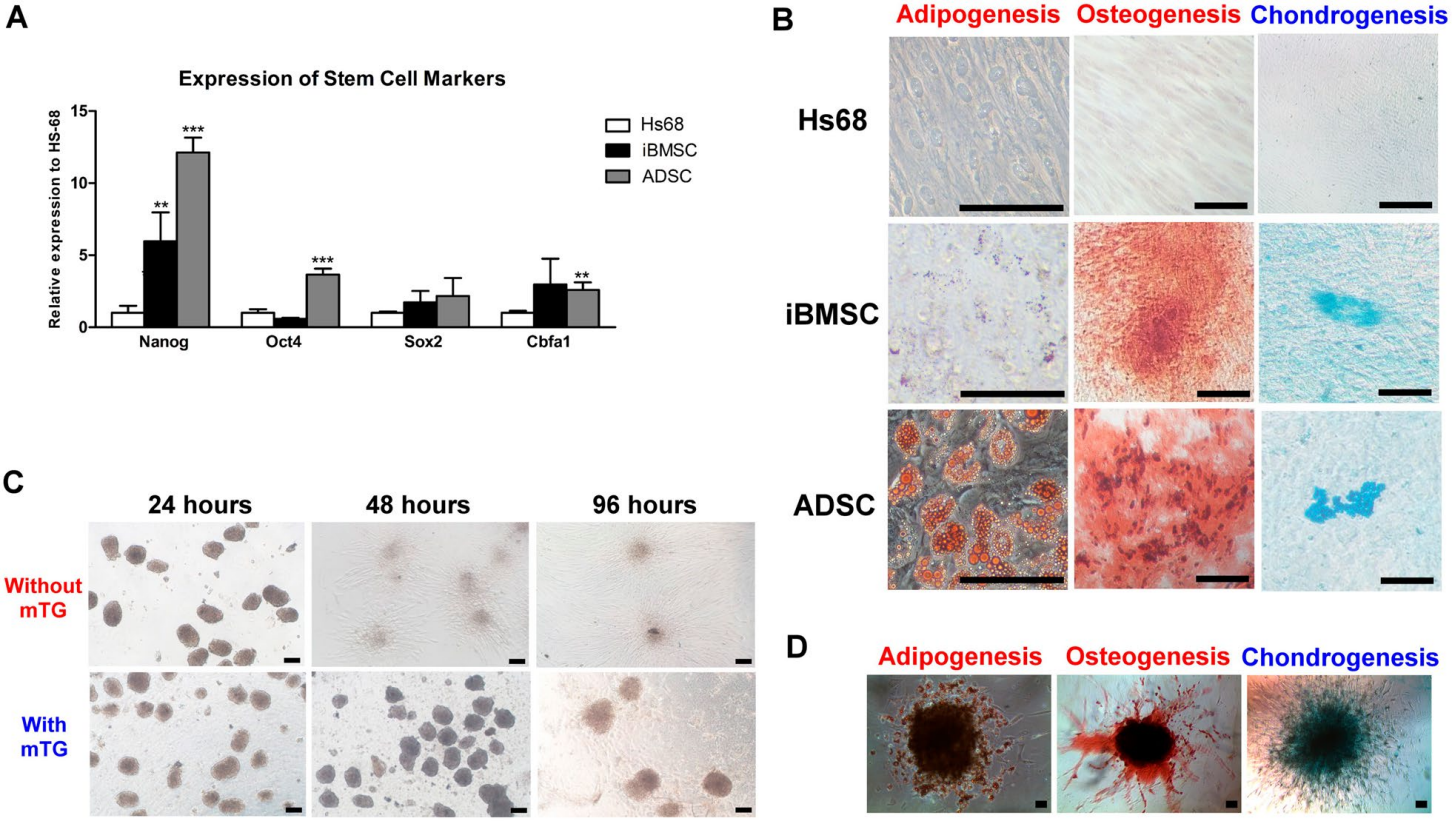
In vitro characterization of ADSCs and ADsp-mTG. **A** Pluripotent gene expression in ADSCs after 24 h of seeding. **B** The trilineage differentiation of ADSCs by Oil Red O (adipogenesis), Alizarin Red (osteogenesis), and Alcian Blue (chondrogenesis) at day 14. Scale bar: 100 µm. **C** The spreading of ADSCs from spheroids embedded in mTG. **D** The trilineage differentiation of ADsp in mTG at day 14. Scale bar: 100 µm. There is no downstream processing or averaging to adjust the resolution of the microscopic images. (Significant difference compared to Hs68: ^*^p < 0.05, ^**^p < 0.01, ^***^p < 0.001)

The expression levels of Nanog, Oct-4, and Cbfa1 were significantly higher in ADSCs than in Hs68 (Fig. 2A). After an induction period of 14 days, there was no sign of adipogenesis, osteogenesis, or chondrogenesis in the cultures of Hs68. In the adipoinductive cultures, lipid droplets slightly accumulated in iBMSCs but obviously accumulated in ADSCs (Fig. 2B). Mineralized nodules and cartilage-specific proteoglycans were deposited in osteoinductive and chondroinductive cultures of iBMSCs and ADSCs.

### Characterization of ADsp-mTG

ADsp successfully formed with an area and Feret diameter of 0.0051 ± 0.0010 mm^2^ and 0.093 ± 0.010 mm, remaining spheroid in shape for 24 h, with an area and Feret diameter of 0.0073 ± 0.0025 mm^2^ and 0.119 ± 0.029 mm without mTG and 0.0059 ± 0.0023 mm^2^ and 0.105 ± 0.024 mm with mTG (Fig. 2C). Without the support of mTG, ADsp spread widely at 48 h. Conversely, when ADsp was encapsulated in mTG, the shapes were maintained for 96 h (Fig. 2C; lower panel), with an area and Feret diameter of 0.0051 ± 0.0015 mm^2^ and 0.099 ± 0.015 mm at 48 h and 0.0055 ± 0.0017 mm^2^ and 0.103 ± 0.016 mm at 96 h. Furthermore, successful adipo-, osteo-, and chondro-induction were noted in ADsp-mTG at day 14 (Fig. 2D).

### The induction of diabetes in rats

**Fig. 3:**
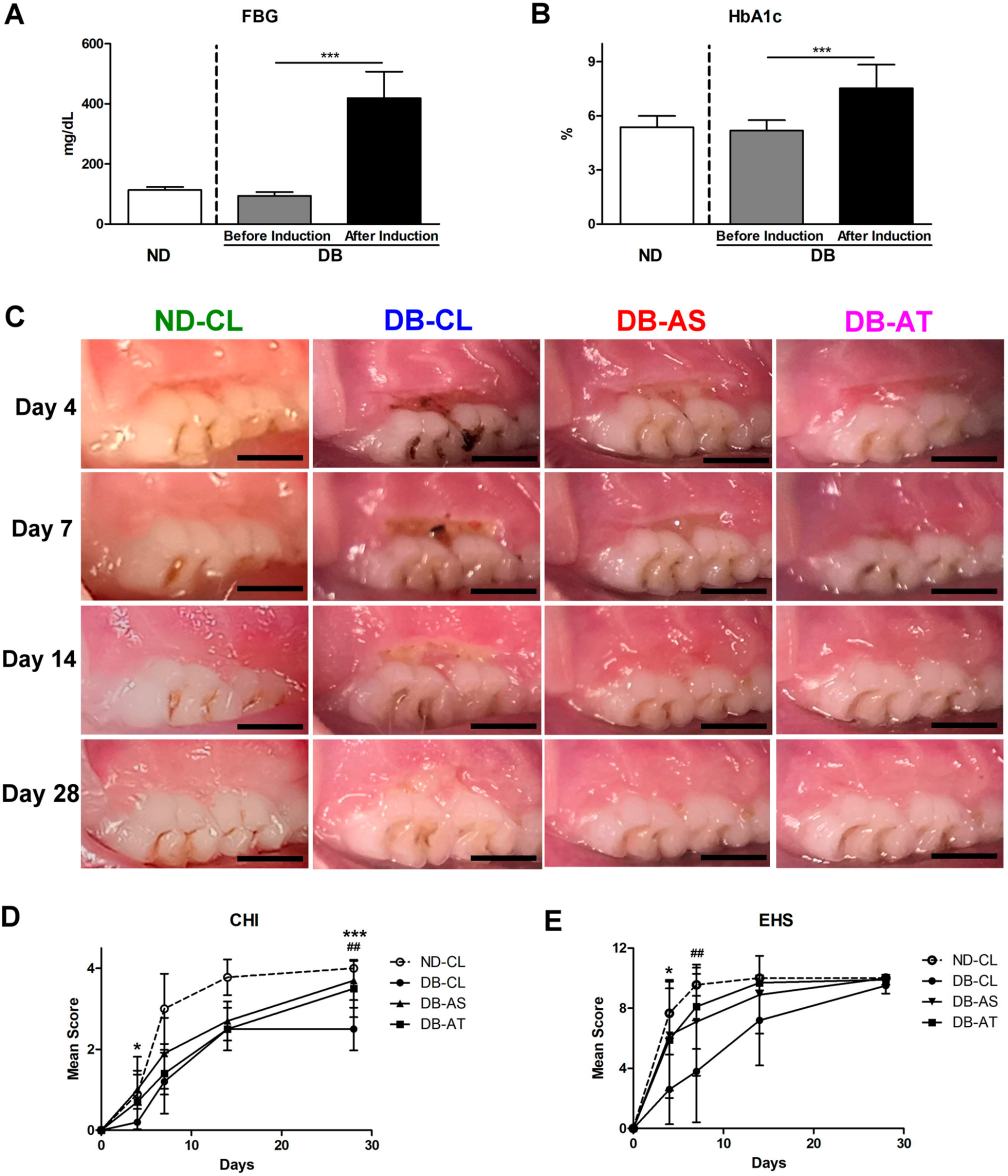
Gross observation of diabetic mucosal wound healing within 28 days. **A** Fasting plasma glucose (FBG) of ND and DB rats. **B** Fasting plasma glucose (FBG) of ND and DB rats. **C** Representative photographs of mucosal wounds. Scale bar: 2 mm. **D** mCHI (range 0–4). **E** mEHS (range 0–10). (In (**A**, **B**), significant difference compared to the same animals before diabetic induction: ^***^p < 0.001; in (**D**, **E**), significant difference between DB-CL and DB-AS group: ^*^p < 0.05, ^***^p < 0.001; significant difference between DB-CL and DB-AT group: ^##^*p* < 0.01)

In DB rats, FBG and HbA1c were 93.58 ± 13.34 mg/dL and 5.18 ± 0.58% before STZ injection and increased significantly to 419.27 ± 87.83 mg/dL and 7.53 ± 1.31% after 3 weeks of STZ injection (p < 0.001 for both; Fig. 3A, B). In ND rats, FBG and HbA1c were 113.20 ± 10.12 mg/dL and 5.37 ± 0.62% at the time of wound/defect creation.

### The effect of ADsp-mTG on diabetic oral mucosal wounds

#### Gross observation

In ND-CL group, wounds were occupied with newly formed tissue at day 4 and were apparently invisible from day 7. Wound dehiscence was noted until day 14 in DB-CL group and until day 7 in DB-AS group. In DB-AT group, wounds were covered with reddish tissue at day 4 and became invisible from day 14 (Fig. 3C).

ND-CL group showed higher scores in CHI and EHS than all groups in DB rats at days 7–14 (Fig. 3D, E). Among DB rats, compared with DB-CL group, CHI in DB-AS and DB-AT groups was greater, specifically at days 4 and 28 (Fig. 3D), and EHS in DB-AS and DB-AT groups was also greater at days 4–14 (Fig. 3E).

#### Histologic and immunohistochemical assessments

**Fig. 4:**
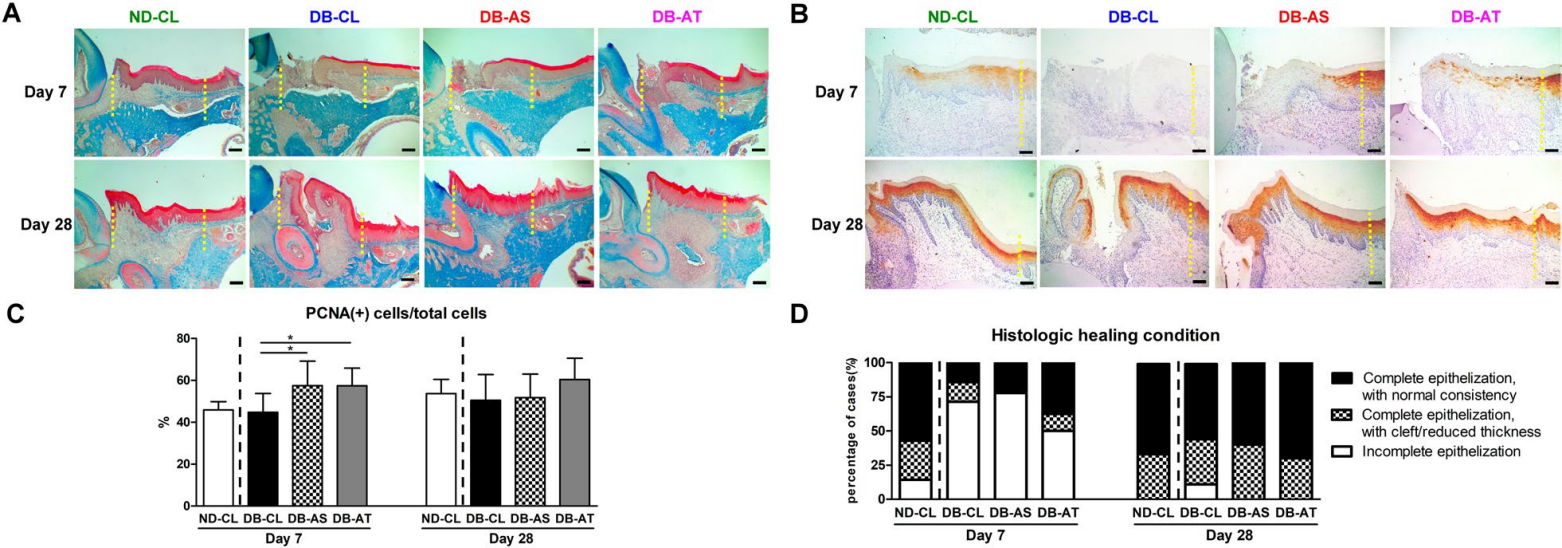
Histologic and immunohistochemical assessments of diabetic mucosal wound healing at days 7 and 28. **A** Representative images of Masson’s trichrome staining. Magnification: 40 × . Scale bar: 250 µm. **B** Representative images of immunohistochemical staining for cytokeratin-10 (K-10). Magnification: 100 × . Scale bar: 100 µm. **C** The ratio of proliferating cells (PCNA( +) cells) in the mucosal wound. **D** The histologic healing condition was categorized into three levels: incomplete epithelization (level 1), complete epithelization with epithelial cleft or reduced connective tissue thickness (level 2), and complete epithelization with normal consistency (level 3). Dashed lines indicate the edge of mucosal wounds. There is no downstream processing or averaging to adjust the resolution of the microscopic images. (Among groups with diabetes, significant difference compared to DB-CL: ^*^p < 0.05, ^**^p < 0.01, ^***^p < 0.001)

At day 7, wound healing was almost complete in the ND-CL group (Fig. 4A). In DB rats, inflammatory cell infiltration along with necrotic tissue adjacent to the tooth was noted. Compared with DB-CL group, the area occupied by necrotic tissue was smaller, and collagen bundle formation beneath the epithelium apparently increased in DB-AS and DB-AT groups. At day 28, the necrotic tissue disappeared, and epithelization was complete in all groups. However, the cleft of the epithelium and sequestrum, with low levels of inflammation, was occasionally seen in DB-CL, DB-AS, and DB-AT groups.

At day 7, K-10, a marker of mucosal keratinization, was not expressed in DB-CL group but was expressed at the border of the wound in DB-AS group and was scattered toward the tooth surface in the epithelium of DB-AT group (Fig. 4B). At day 28, K-10 was evenly distributed in the outer epithelium but was not expressed at the sulcular epithelium or the bottom of the epithelial cleft, regardless of the treatment group (Fig. 4B). Compared with DB-CL group, the ratio of proliferating cells significantly increased in DB-AS and DB-AT groups at day 7 and slightly increased in DB-AT group at day 28 (Fig. 4C).

The categorized histologic healing condition is shown in Fig. 4D. At day 7, epithelization was seen in most specimens of ND-CL group but wound dehiscence was generally noted in DB rats. Among DB rats, epithelization was more commonly noted in DB-AT group. At day 28, although wound dehiscence without coverage of the epithelium was still noted in one specimen from DB-CL group, complete epithelization with adequate lamina propria formation was seen in > 50% of specimens in all groups.

### The effect of ADSC-mTG on diabetic osseous defects

#### The osteogenic differentiation level of pretreated ADSCs in vitro

**Fig. 5:**
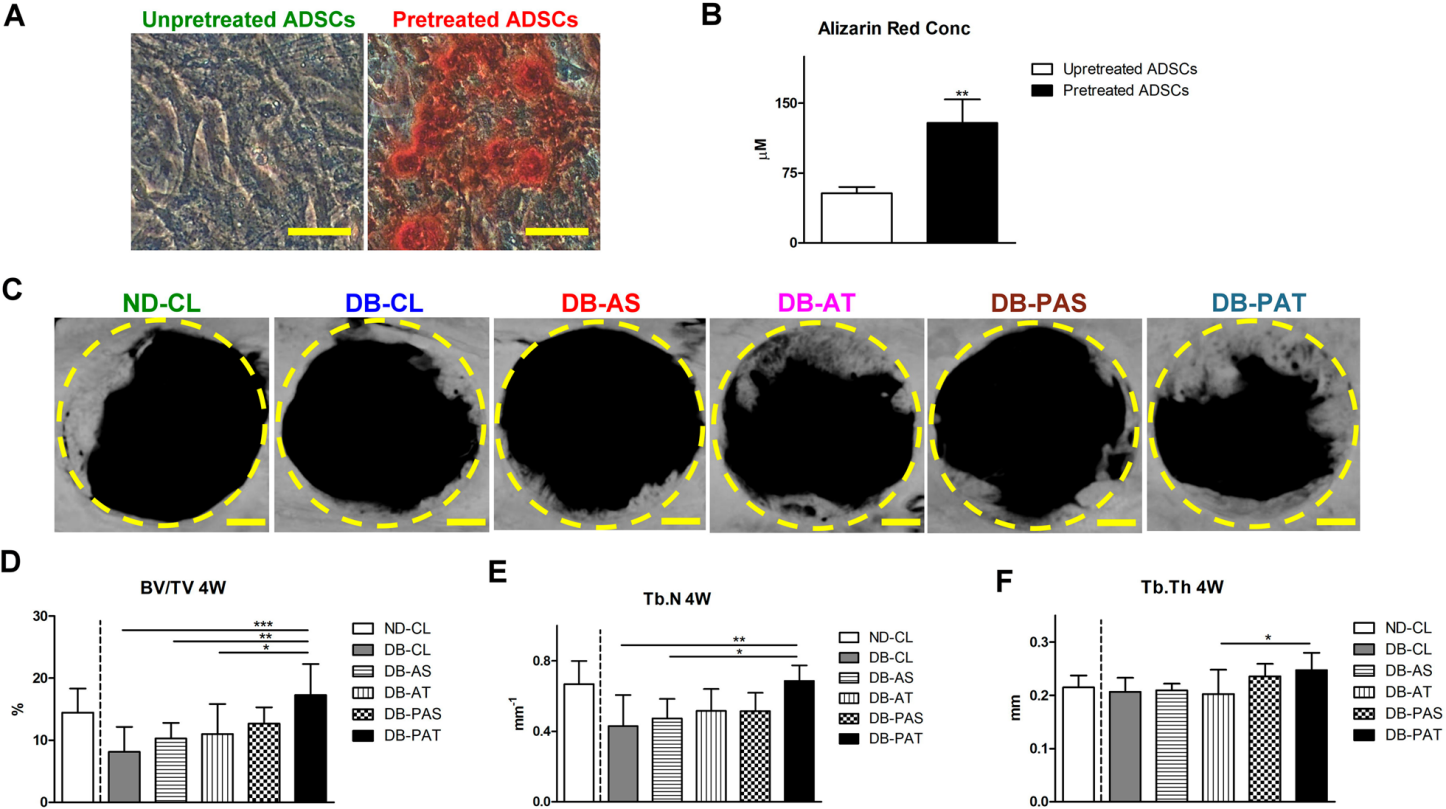
Osteogenic differentiation of ADSCs after 10 days pretreatment in vitro and the micro-CT assessments of diabetic osseous defect healing at day 28. **A** Mineralized matrix deposition by Alizarin Red. Scale bar: 50 µm. **B** The concentration of Alizarin Red. **C** Representative slices from the images. Scale bar: 1 mm **D** Percentage bone volume (BV/TV) Tb.N. **F** Tb.Th. Dashed lines indicate the borders of osseous defects. (In (**B**), significant difference compared to the unpretreated ADSCs: **p < 0.01; in (**D**-**F**), among groups with diabetes, significant difference compared to DB-CL: ^*^p < 0.05, ^**^p < 0.01, ^***^p < 0.001)

Compared with the cultures of unpretreated ADSCs, mineralized matrix was evidently deposited, and the concentration of Alizarin Red was significantly greater, in the cultures of pretreated ADSCs (p < 0.01; Fig. 5A, B).

#### Micro-CT assessments

NB in the defects was not visualized at day 7 in all groups (data not shown). At day 28, NB was noted on the defect borders in all groups (Fig. 5C). Compared with the ND-CL group, BV/TV and Tb.N were inferior in DB-CL group (Fig. 5D, E). Among DB rats, BV/TV was significantly greater in DB-PAT group than in DB-CL, DB-AS, and DB-AT groups (Fig. 5D); Tb.N was significantly greater than that in DB-CL and DB-AS groups (Fig. 5E); and Tb.Th was significantly greater than that in DB-AS group (Fig. 5F).

#### Histologic and immunohistochemical assessments

**Fig. 6:**
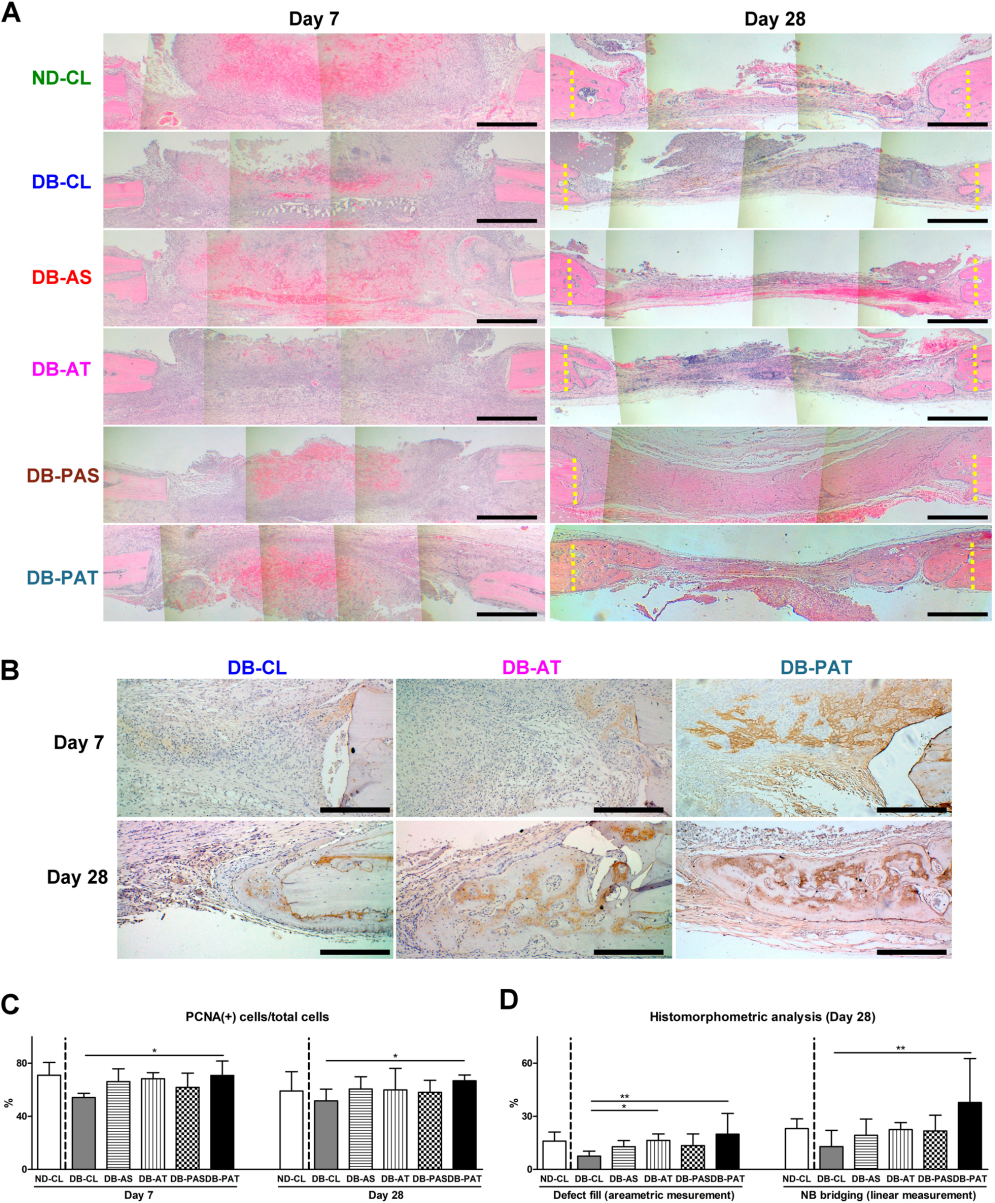
Histologic and immunohistochemical assessments of diabetic osseous defect healing at days 7 and 28. **A** Representative images of hematoxylin and eosin staining. Magnification: 40 × . Scale bar: 1 mm. **B** Representative images of immunohistochemical staining for bone sialoprotein (BSP). Magnification: 100 × . Scale bar: 100 µm. **C** The ratio of proliferating cells (PCNA( +) cells) in the osseous defect at days 7 and 28. **D** Histomorphometric analysis of the defect fill and new bone (NB) bridging at day 28. Dashed lines indicate the borders of osseous defects. There is no downstream processing or averaging to adjust the resolution of the microscopic images. (Among groups with diabetes, significant difference compared to DB-CL: ^*^p < 0.05, ^**^p < 0.01, ^***^p < 0.001)

At day 7, the defects were mainly occupied with nonmineralized granulation tissue in all groups (Fig. 6A). BSP, an early osteogenic marker, was lightly deposited at the defect peripheries in DB-CL, DB-AS, DB-AT, and DB-PAS groups and was widely spread in DB-PAT group (Fig. 6B, and see Appendix Figure for the images of DB-AS and DB-PAS groups). Compared with DB-CL group, the ratio of proliferating cells was significantly greater in DB-PAT group at days 7 and 28 (Fig. 6C). At day 28, NB formation from the peripheries toward the center of the defect was notable in all groups (Fig. 6A). Although the closure of the defect did not take place in any of the specimens, NB formation was more prominent in DB-PAT groups among all groups. BSP was deposited within NB, with heavier deposition in DB-PAT group (Fig. 6B). Compared with DB-CL group, defect filling was significantly greater in DB-AT and DB-PAT groups, and NB bridging was significantly greater in DB-PAT group (Fig. 6D).

## Discussion

The present study demonstrated the stemness of ADSCs based on the pluripotent gene expression and trilineage differentiation capability (Fig. 2A-B). According to a previous study, encapsulating ADSC spheroids (ADsp) in mTG provided favorable injectability for delivering ADsp [18]. This configuration also maintained the 3D configuration to mimic the in vivo microenvironment and the differentiation properties of ADSCs (Fig. 2C, D), thereby increasing the regenerative potential for therapeutic use [15, 18]. These results supported ADsp-mTG as a good candidate for promoting the healing of mucosal wounds and osseous defects.

A previous study reported that keratinocyte and fibroblast migration and proliferation were impaired in diabetic animals [24], and similar outcomes were noted in the mucosal wounds of DB-CL group in the present study (Fig. 4B, C). ADSCs have been recommended to treat diabetic wounds according to their multilineage differentiation, chemotaxis promotion, extracellular matrix synthesis, and anti-inflammation capabilities [25]. While ADsp-mTG maintained the 3D configuration of cellular aggregates and stemness and improved the handling property and kept ADSCs in place, ADsp-mTG delivery further facilitated wound healing, and keratinization was noted in diabetic mucosal wounds (Figs. 3 and 4).

The retardation of wound repair in diabetic animals was also noted in the osseous defects (Figs. 5 and 6). However, applications of ADsp (i.e., DB-AS and DB-AT groups) showed limited effects (Figs. 5 and 6D), potentially due to the effects of diabetes on the activities of stem cells. Previous studies reported that stem cells from the diabetic environment presented inferior osteogenic and angiogenic potential but were apparently more adipogenic [26, 27]. Stem cells incubated with AGEs or in a high-glucose environment also exhibited reduced osteogenic ability [28, 29]. Due to the unfavorable osteogenic environment and lack of osteogenic signals in diabetes, pretreatment of ADSCs to direct differentiation toward osteogenesis may be required for treating diabetic osseous defects. In DB-PAT group, BSP, an osteogenic marker, was highly expressed at the early stage, whereby such distinction was not seen in DB-CL and DB-AT groups (Fig. 6B), and NB formation was significantly greater than that in DB-CL group at the later stage (Fig. 6). It is noteworthy that NB formation and defect fill in DB-PAT group were even greater than those in ND animals (Figs. 5 and 6). Taken together, pretreated ADsp-mTG recovered the osteogenic impairment influenced by diabetes and could be a viable application for treating diabetic osseous defects.

This study has limitations. First, STZ destroyed pancreatic islet β-cells and induced mostly type I DM in this study [30], but type II DM accounts for approximately 90% of clinical DM [1]. While FBG and HbA1c were significantly elevated following STZ injection, hyperglycemia and AGE disturbance of DB animals still caused comorbidities similar to clinical DM. Second, because the creation of a critical-sized periodontal defect in rats similar to that in humans is impractical, we assessed the mucosal and bony compartments separately by using oral mucosal wounds and calvarial osseous defects. However, as the calvarium develops differently from jaw bones [31] and the etiology of defect generation is different from periodontal defects, the results from this study should be interpreted carefully. Third, without protection from wound dressings or adhesives, mTG may be washed out rapidly in mucosal wounds to attenuate the influence of ADSCs. Furthermore, the osseous defect fill in this study was obviously lower than that in other relevant animal studies [32–34], and the potential reason was the lack of the osteoconductive framework provided by the scaffold. The combination of ADsp with osteoconductive scaffolds, such as 3D-printed hydroxyapatite-based scaffolds [35], may be beneficial to promote the efficiency of ADsp-mTG for osseous defect regeneration. Further investigations in more clinically relevant conditions, with adequate dressing/adhesive materials and osteoconductive scaffolds, are still necessary.

## Conclusion

ADsp-mTG showed trilineage differentiation capability. ADsp-mTG accelerated diabetic oral mucosal wound healing, and osteogenically pretreated ADsp-mTG promoted diabetic calvarial osseous defect regeneration, validating the concept of ADsp-mTG application for facilitating the healing of diabetic periodontal wounds and craniofacial defects.

## Figures and Tables

**Fig. 7:**
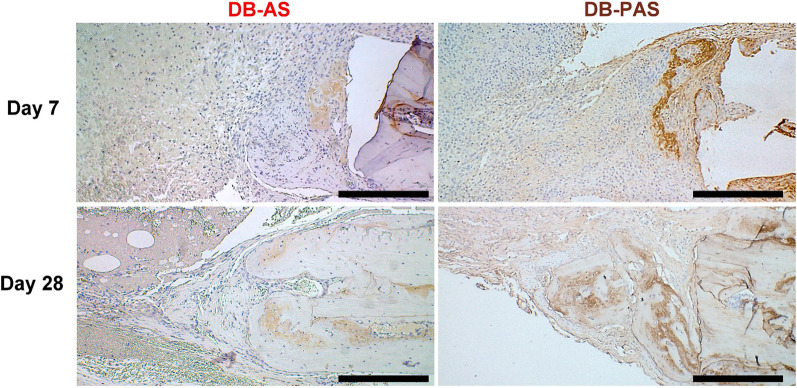
Representative images of immunohistochemical staining for bone sialoprotein (BSP) in DB-AS and DB-PAS groups. Magnification: 100 × . Scale bar: 100 µm

## Data Availability

The data generated and analyzed in this study are available from the corresponding author upon reasonable request.

## Appendix

### Manufacturer information of materials

**Table:**

Name	Manufacturer information
iBMSC (cell line)	Applied Biological Materials Inc., Richmond, BC, Canada
Hs68 (cell line)	Bioresource Collection and Research Center, Hsinchu, Taiwan
Fetal bovine serum	Thermo Fisher Scientific Co., Waltham, MA, USA
Penicillin–streptomycin	Thermo Fisher Scientific Co., Waltham, MA, USA
Microbial transglutaminase	Moo Gloo™; Modernist Pantry, LLC., Eliot, ME, USA
Zolazepam-tiletamine	Zoletil 50; Virbac, Cedex, France
Xylazine	Rompun 20; Bayer Animal Health, Monheim, Germany
RNA isolation kit	RNeasy Mini Kit; QIAGEN GmbH, Hilden, Germany
cDNA synthesis kit	Bio-Rad Laboratories, Hercules, CA, USA
Master Mix	Thermo Fisher Scientific Co., Waltham, MA, USA
Polyclonal antibody for K-10	Applied Biological Materials Inc., Richmond, BC, Canada
Polyclonal antibody for PCNA	Proteintech Group, Inc., Rosemont, IL, USA
Polyclonal antibody for BSP	Thermo Fisher Scientific Co., Waltham, MA, USA
Cell and tissue staining kit	Super Sensitive™ Polymer-HRP Detection Kit; Bio-Genex Laboratories, Fremont, CA, USA
Reflex wound clips	MikRon Precision Inc., Gardena, CA, USA

*Materials not listed in the table were all from Sigma-Aldrich, St Louis, MO, USA.

### Manufacturer information of equipment

**Table:**

Name	Manufacturer information
Real-time PCR system	ABI 7800; Applied Biosystems, Foster, CA, USA
Micro-CT scanner	SkyScan1176; Bruker Corp., Kontich, Belgium
Micro-CT image analysis software	CTAn; Bruker Corp., Kontich, Belgium
Digital camera	LM-K520YMW; LG Electronics Inc., Seoul, Korea
Light microscope (with lens)	Leica DM500; Leica Microsystems, Wetzlar, Germany
Digital imaging system (with camera and software)	AxioCam ICc5; Carl Zeiss Microscopy GmbH, Munich, Germany
ImageJ	NIH, Bethesda, MA, USA
Glucose meter	Accu-Chek® Instant, Roche, Indianapolis, IN, USA
HbA1c analyzer	Eclipse Arc™, Apex BioTech Co., Hsinchu, Taiwan
ELISA reader	Biotek Synergy HT microplate reader; Agilent Technologies Inc., Santa Clara, CA, USA
Statistical software	GraphPad Prism®; GraphPad Software Inc., San Diego, CA, USA

### Formulations of medium

**Table:**

Medium	Formulation
Growth medium	DMEM/F12 supplemented with 1 ng/mL basic fibroblast growth factor for ADSC, PriGrow II medium (Applied Biological Materials Inc.) for iBMSC and HS-68
Adipogenic induction medium	DMEM supplemented with 10% FBS, 1% penicillin/streptomycin, 500 µM 3-isobutyl-1-methylxanthine, 1 µM dexamethasone, 10 µM insulin and 200 µM indomethacin
Osteogenic induction medium	DMEM supplemented with 10% FBS, 1% penicillinestreptomycin,10 nM dexamethasone, 50 µM ascorbic acid 2-phosphate, and 10 µM β-glycerophosphate
Chondrogenic induction medium	DMEM supplemented 1% FBS, 1% PSA, 10 ng mL^−1^ transforming growth factor beta-1, 50 mM L-ascorbic acid-2-phosphate and 6.25 mg mL^−1^ insulin

### Clinical healing index (CHI)

**Table:**

Degree	Description
0	< 1/3 Epithelization
1	1/3 – 2/3 Epithelization
2	> 2/3 Epithelization, but not fully epithelization
3	Fully epithelization, reduced in height and redder than normal
4	Fully epithelization, normal color, consistency, surface texture, and contour

### Early wound healing score (EHS)

**Table:**

Parameter	Point	Description
Clinical signs of re-epithelization (CSR)	0	Visible distance between incision margins
	3	Contact between incision margins
	6	Merged incision margins
Clinical signs of hemostasis (CSH)	0	Bleeding at the incision margins
	1	Presence of fibrin on the incision margins
	2	Absence of fibrin on the incision margins
Clinical signs of inflammation(CSI)	0	Redness involving > 50% of the incision length and/or pronounced swelling
	1	Redness involving < 50% of the incision length
	2	Absence of redness along the incision length

The EHS score is the sum of CSR, CSH and CSI.

### The conditions of real-time PCR

cDNA mixed with the TaqMan probes and Master Mix to achieve a volume of 30 µl, and real-time PCR was performed using a real-time PCR system under the following conditions: hot-start enzyme activation at 95 °C for 3 min, followed by 40 cycles of denaturation at 95 °C for 10 s and 60 °C for 40 s.

### The list of TaqMan probes used for real-time PCR

**Table:**

	Manufacturer	Reference Seqeunce	Assay ID
GAPDH	Thermo Fisher Scientific	NM_017008.4	Rn01462662_g1
Oct4	Thermo Fisher Scientific	NM_001009178.2	Rn06413993_s1
Sox2	Thermo Fisher Scientific	NM_001109181.1	Rn01286286_g1
Cbfa1	Thermo Fisher Scientific	NM_001278483.1	Rn01512298_m1
Nanog	Thermo Fisher Scientific	NM_001297698.1	Hs02387400_g1

### The protocol of the immunohistochemical staining

The immunohistochemical staining was performed using a cell and tissue staining kit following the manufacturer’s instructions. In brief, antigens and epitopes were unmasked by incubation with 0.05% trypsin/EDTA for 20 min at room temperature. To eliminate endogenous peroxidase activity, the sections were immersed in 3% H_2_O_2_ for 10 min. After blocking of non-specific binding with serum, the sections were incubated overnight at 4 °C with the rabbit polyclonal anti-K-10 (1:1000 dilution), anti-PCNA (1:200 dilution), or BSP (1:200 dilution). The sections were subsequently incubated with the corresponding biotinylated secondary antibodies for 1 h at room temperature. The color was developed by 3,3-diaminobenzidine, and sections were counterstained with hematoxylin (Fig. 7).

## Abbreviations

ADSC: Adipose-derived stem cell
ADsp: ADSC spheroids
mT: Microbial transglutaminase
mTG: MT cross-linked gelatin hydrogel
DM: Diabetes mellitus
NTUH: National Taiwan University Hospital
Sox2: SRY-box transcription factor-2
Oct4: Octamer-binding transcription factor-4
Cbfa1: Core-binding factor alpha-1
Hs68: A human foreskin fibroblast cell line
iBMSC: Induced bone marrow mesenchymal stem cell line
STZ: Streptozotocin
FBG: Fasting blood glucose
HbA1c: Glycated hemoglobin
DB: Diabetes induction
ND: Non-diabetic
DB-CL: Untreated control sites in DB animals
DB-AS: ADsp-treated sites in DB animals
DB-AT: ADsp-mTG-treated sites in DB animals
ND-CL: Untreated control sites in ND animals
CHI: Clinical healing index
EHS: Early wound healing score
K-10: Cytokeratin-10
PCNA: Proliferating cell nuclear antigen
DB-PAS: Osteogenically pretreated ADsp-treated sites in DB animals
DB-PAT: Osteogenically pretreated ADsp-mTG-treated sites in DB animals
BV/TV: Relative bone volume (bone volume/tissue volume)
Tb.Th: Trabecular thickness
Tb.N: Trabecular number
NB: New bone
